# Retrospective Cross-Sectional Study of the Relationship of Thyroid Volume and Function with Anthropometric Measurements, Body Composition Analysis Parameters, and the Diagnosis of Metabolic Syndrome in Euthyroid People Aged 18–65

**DOI:** 10.3390/medicina60071080

**Published:** 2024-06-30

**Authors:** Grzegorz K. Jakubiak, Natalia Pawlas, Małgorzata Morawiecka-Pietrzak, Monika Starzak, Agata Stanek, Grzegorz Cieślar

**Affiliations:** 1 Department of Internal Medicine, Angiology and Physical Medicine, Faculty of Medical Sciences in Zabrze, Medical University of Silesia, 41-902 Bytom, Poland; astanek@tlen.pl (A.S.); cieslar1@tlen.pl (G.C.); 2 Department of Pharmacology, Faculty of Medical Sciences in Zabrze, Medical University of Silesia, 41-800 Zabrze, Poland; n-pawlas@wp.pl; 3 Department of Pediatrics, Faculty of Medical Sciences in Zabrze, Medical University of Silesia, 41-800 Zabrze, Poland; morawieckam@gmail.com; 4 Department of Internal Medicine, Angiology and Physical Medicine, Specialist Hospital No. 2, 41-902 Bytom, Poland; mslekarz@gmail.com

**Keywords:** thyroid volume, thyrotropin, triiodothyronine, thyroxine, body composition analysis, overweight, obesity, metabolic syndrome

## Abstract

*Background and Objectives*: The thyroid is a key endocrine gland for the regulation of metabolic processes. A body composition analysis (BCA) is a valuable complement to the assessment of body mass index, which is derived only from body weight and height. This cross-sectional retrospective study aimed to investigate the relationships between thyroid volume (TV) and thyroid function parameters, anthropometric measurements, BCA parameters, and the presence of metabolic syndrome (MetS) in adults without clinically overt thyroid disease. *Material and Methods*: This study involved 45 people (females: 57.8%; MetS: 28.9%) hospitalized for planned diagnostics without signs of acute illness or a deterioration of their health and without thyroid disease, who underwent thyroid ultrasound scans, biochemical tests to assess their thyroid function, MetS assessments, anthropometric measurements, and BCAs using the bioelectrical impedance method. *Results:* The TV was significantly larger in people with MetS compared to people without MetS. The TV was significantly higher and the serum thyrotropin (TSH) concentration was significantly lower in overweight and obese people than in normal and underweight people. The free triiodothyronine (FT3) serum concentration and TV were correlated with waist circumference and some parameters of the BCA, and the FT3 concentration was also correlated with the body mass index, waist–hip ratio, and waist–height ratio. No significant correlations were found between the FT4 and TSH and the results of the anthropometric and BCA measurements. *Conclusions:* Even in a population of euthyroid patients without clinically overt thyroid disease, there were some significant relationships between the volume and function of the thyroid gland and the results of their anthropometric parameters, BCAs, and the presence of MetS features.

## 1. Introduction

Obesity is becoming one of the most serious health problems in many countries around the world [1]. Obesity predisposes people to the development of metabolic syndrome (MetS), which is a set of abnormalities, such as hyperglycemia or type 2 diabetes mellitus (T2DM), arterial hypertension, and atherogenic dyslipidemia. These significantly increase the risk of developing atherosclerotic cardiovascular diseases (CVDs) [2], which are one of the most significant causes of morbidity and mortality worldwide [3]. The basic components of MetS include abdominal obesity, hyperglycemia, decreased high-density lipoprotein cholesterol, increased triglycerides, and increased blood pressure [4]. Additional pathologies that often occur in the course of MetS, although they are not part of its diagnosis criteria, include metabolic fatty liver disease, hyperuricemia, tachycardia, left ventricular myocardial hypertrophy, heart failure with preserved left ventricular ejection function, and obstructive sleep apnea [5]. Obesity and MetS are associated with increased oxidative stress [6,7], which plays an important role in the pathogenesis of numerous diseases, such as CVDs, cancers, and neurodegenerative diseases. Therefore, improving our knowledge of the pathophysiology of obesity and related abnormalities plays an important role.

The basic tool that allows for diagnoses of obesity in clinical practice is the body mass index (BMI), which is defined as the ratio of one’s weight, expressed in kilograms, to the square of one’s height, expressed in meters. Although this parameter is simple, widely available, and easy to use and interpret, it has a number of limitations. First of all, it does not refer to body composition at all, especially fat content and its distribution [8]. Moreover, an excessive accumulation of visceral adipose tissue and its dysfunction play key roles in the pathogenesis of obesity-related metabolic disorders [9]. A body composition analysis (BCA) can, therefore, be a valuable complement to the BMI. In clinical practice, the bioelectrical impedance method is widely used for BCAs [10].

The thyroid is a small organ located in the front of the neck, composed of two lobes and an isthmus. Histologically, the thyroid parenchyma is composed of vesicles containing a glycoprotein called thyroglobulin, in which thyroid hormones (thyroxine and triiodothyronine) are stored. The thyroid gland function is regulated by negative feedback from the thyrotropin synthesized in the anterior pituitary gland [11]. Controlling the rate of metabolic processes is a key function of thyroid hormones. However, this issue is complex. It is commonly known that changes in body weight are involved in the typical clinical picture of thyroid dysfunction. On the other hand, there are research results that show that overweight and obesity may lead to an increase in thyrotropin concentration in the blood [12,13,14,15]. The study of BCA has already attracted the interest of researchers in patients with overt thyroid disease [16,17,18]. Therefore, it is interesting to see whether there is a relationship between the metabolic and anthropometric parameters, including BCAs, and the parameters of thyroid morphology and function in the case of people without thyroid function disorders.

The purpose of this study was to investigate the relationship between the parameters of thyroid function assessment and the results of anthropometric measurements and BCAs, as well as the diagnosis of MetS in people aged 18–65 years without clinically overt thyroid function abnormalities.

## 2. Materials and Methods

### 2.1. Study Population

This study consisted of a retrospective analysis of data from the medical records of patients hospitalized in the Department of Internal Medicine, Angiology, and Physical Medicine of the Medical University of Silesia from June 2022 to October 2023. This study included only people aged 18 to 65 years who, during hospitalization, underwent BCAs, thyroid ultrasound scans, and tests of their basic biochemical parameters, including their thyroid function parameters, blood glucose, and lipid profile. People with heart failure, water–mineral balance disturbances, infection, or any acute illness or exacerbation of chronic disease within the month preceding their admission to the hospital were excluded. This study did not include patients with diagnosed thyroid disease, including those using levothyroxine replacement or thyrostatic treatment, even if they were euthyroid according to the current test results.

### 2.2. Laboratory Tests

Blood was collected for basic laboratory tests in the morning between 8 and 10 a.m., at least fourteen hours after the patient’s last meal. Thyroid function was assessed by measuring the serum concentrations of thyrotropin (TSH), free triiodothyronine (FT3), and free thyroxine (FT4).

### 2.3. Thyroid Ultrasound

Each patient underwent thyroid ultrasound scans using a Samsung device (RS80 EVO) with a linear probe (LA4-18B). In each case, the examination was performed by the same physician, who was experienced in performing this diagnostic procedure. In each examination, the location of the thyroid gland, the echogenicity of the thyroid parenchyma, the sizes of both lobes (in three dimensions), and the thickness of the isthmus were assessed. Based on the dimensions of the thyroid lobes, the estimated volume of the gland was calculated. Additionally, the thyroid gland was assessed for the presence of possible focal lesions, which were described in each case in terms of their size and structure.

### 2.4. Body Composition Analyses (BCAs) and Basic Anthropometric Parameters

BCAs and anthropometric measurements were performed in the morning, before the patient’s first meal, between 7 and 9 a.m.

BCAs were performed using the bioelectrical impedance method employing a TANITA MC-780 apparatus. The test results included parameters such as body mass, body mass index, fat mass and percentage, fat-free mass, muscle mass and percentage, bone mass, skeletal muscle mass, metabolic age, visceral fat rating, total body water (mass and percentage), extracellular-to-total body water ratio, and basal metabolic rate.

In addition, each patient’s height, waist circumference, and hip circumference were measured. Based on the measured parameters, the waist–hip ratio (WHR) and the waist–height ratio (WtHR) were calculated.

### 2.5. Diagnosis of Metabolic Syndrome (MetS)

The diagnosis of MetS was based on the definition of the joint position of the International Diabetes Federation, National Heart, Lung, and Blood Institute, American Heart Association, World Heart Federation, International Atherosclerosis Society, and International Association for the Study of Obesity [19]. The diagnosis of MetS was made in individuals who met at least three of the five criteria presented in Table 1.

### 2.6. Statistical Analysis

The compliance of the distribution of the quantitative variables with the normal distribution was examined using the Shapiro–Wilk test, analysis of the distribution parameters, and visual assessment of the histogram.

The values of the quantitative variables were presented as the mean and standard deviation (SD) for those whose distribution did not differ significantly from the normal distribution. The values of the quantitative variables were presented as the median and the first and the third quartiles values (Q1; Q3) for those whose distribution differed significantly from the normal distribution. Ranges were also provided for all quantitative variables. The values of the qualitative variables were presented in the form of the number of a given variant and the percentage.

Spearman’s rank correlation test was used to examine the correlation between the anthropometric and BCA parameters and the thyroid morphology and function parameters. To compare the significance of differences among the subgroups, Student’s *t*-test was used for variables whose distribution did not differ significantly from the normal distribution, and the Mann–Whitney U test was used for variables whose distribution differed significantly from the normal distribution. *p* < 0.05 was considered statistically significant.

To compare the values of the thyroid assessment parameters among subgroups based on BMI category, one-way ANOVA was used, or, if the assumptions were not met, the Kruskal–Wallis test was used. The homogeneity of variances was tested using Levene’s test. *p* < 0.05 was considered statistically significant.

Statistical analysis was performed using TIBCO Software Inc. (Palo Alto, CA, USA, 2017) Statistica (data analysis software system), version 13.

### 2.7. Ethical Aspects

An inquiry was submitted to the Bioethics Committee of the Medical University of Silesia in Katowice, who responded that a study involving a retrospective analysis of medical records does not require approval of the Bioethics Committee (6 February 2024, BNW/NWN/0052/KB/19/24).

## 3. Results

### 3.1. Study Participants: General Characteristics

A total of 45 patients were included in the final analysis, 57.8% of whom were women. The median age was 49.9 years (37.6; 56.2) (N = 45). In the study group, 19 patients (42.2%) had diagnosed hypertension, five patients (11.1%) had T2DM, four patients (8.9%) had prediabetes, and 19 patients (42.2%) had atherosclerosis (one person had undergone a percutaneous coronary intervention in the past; in the case of the remaining people, only insignificant atherosclerotic plaque was found in their imaging diagnostics, without the diagnosis of atherosclerotic CVD). Among the study participants, 15 (33.3%) had never smoked tobacco, 13 (28.9%) admitted that they currently smoked tobacco, and 16 (35.6%) had smoked tobacco in the past. None of the study population abused alcohol.

### 3.2. Results of Assessment of Thyroid Volume (TV) and Function

Table 2 presents descriptive statistics of the parameters for assessing thyroid volume (TV) and thyroid function parameters. In one case, the TV was not measured due to the retrosternal location of part of the organ, which prevented its full visualization. In 15 people (33.3%), focal lesions were found, no cases of which had ultrasound features that qualified them for biopsies, only further observation.

### 3.3. Anthropometric Measurements and Body Composition Analysis (BCA)

Table 3 presents the results of the anthropometric measurements and BCA parameters. Table 4 shows the number of individual categories, taking into account the BMI values.

It should be noted that the study participants included people from each BMI category, i.e., underweight people, normal-weight people, overweight people, and obese people. There were no morbidly obese people in the study population because the highest BMI value was equal to 37.5 kg/m^2^. Therefore, the study population was diverse in terms of anthropometric parameters, which is also reflected in the waist circumference and fat percentage values, which varied in wide ranges (61.0–117.0 cm and 7.3–41.0%, respectively).

### 3.4. Correlations

Table 5 presents the results of the Spearman’s rank correlation test among the parameters of the thyroid function assessment, anthropometric parameters, and BCAs.

It was found that the FT3 concentration was significantly and positively correlated with the BMI, waist circumference, WHR, WtHR, fat-free body mass, bone mass, muscle mass, water mass (but not with the percentage), skeletal muscle mass, and basal metabolic rate. The TV was found to be significantly and positively correlated with the waist circumference (but not with the WHR or WtHR), fat-free mass, bone mass, muscle mass, water mass (but not with the percentage), skeletal muscle mass, visceral fat rating, and basal metabolic rate. The strongest correlation was found between the TV and the fat-free body mass, bone mass, and muscle mass, while slightly weaker correlations were found between the TV and the basal metabolic rate, water mass, and skeletal muscle mass.

According to the results obtained, neither the TSH nor FT4 was significantly correlated with the results of the anthropometric measurements or the BCA parameters.

### 3.5. Comparison of Patients with and without Metabolic Syndrome

Among the people without MetS, the percentage of women was 68.75%, and among the people with MetS, the percentage of women was 30.77%.

Table 6 shows the differences in the BCA parameters and thyroid volume and function between people with MetS and people without diagnosed MetS. The people with MetS were significantly older than the people without MetS and had significantly higher BMI, waist circumference, WHR, and WtHR values. However, there were no significant differences in the fat mass or percentage. The people with MetS in the study population had significantly higher values of fat-free body mass, bone mass, and muscle mass (but not percentage). The skeletal muscle mass was significantly higher among the people with MetS. Among the people with MetS, the body water mass was significantly higher in absolute value, but there was no significant difference in percentage. There was no significant difference in the ratio of extracellular to total water. The people with MetS had a significantly higher visceral fat rating, metabolic age, and basic metabolic rate.

The people with MetS had a significantly higher TV value, but no significant differences were found in the biochemical parameters assessing thyroid function.

### 3.6. Thyroid Function Parameters in Subgroups According to Body Mass Index

Table 7 shows a comparison of the TV values and biochemical parameters for the assessment of thyroid function depending on the BMI category (normal weight, overweight, obesity). Due to the small number of underweight people (N = 4), this category was not included in this analysis. There were no significant differences among the individual BMI categories in terms of the TSH, FT3, and FT4 values. Significant differences were found for the TV. The post hoc analysis showed that the difference between the normal-weight and overweight people was significant (*p* = 0.024).

Table 8 presents the results of an additional analysis comparing the volume and parameters of thyroid function between subjects with a BMI of <25.0 kg/m^2^ (underweight and normal-weight patients taken together) and subjects with a BMI of ≥25.0 kg/m^2^ (overweight and obese patients taken together). The median TV was significantly higher and the median serum TSH concentration was significantly lower among the overweight or obese people taken together compared to the underweight people and people with a normal body weight taken together. No significant differences were found in their FT3 and FT4.

It is worth noting that after excluding underweight people, the differences between the TV and the serum TSH concentration remain statistically significant also when comparing normal-weight people with overweight or obese people. Among the underweight people, the BMI values were 16.4 kg/m^2^, 18.09 kg/m^2^, 18.29 kg/m^2^, and 18.64 kg/m^2^, respectively.

## 4. Discussion

In this publication, we present the results of an analysis of the interrelationships between the TV and biochemical parameters for the assessment of thyroid function and some anthropometric parameters, BCAs, and the presence of the MetS. Similar issues have already been studied by other scientists.

We showed that their FT3 and TV were significantly correlated with the values of some anthropometric measurements and the BCAs, and the strongest correlations concerned the TV. In the material we collected, we did not find any significant correlation between the TSH and FT4 values and the values of the anthropometric parameters and BCAs. In the material we collected, the TV differed significantly between people with and without MetS, and in terms of BMI categories, we found a significant difference in the TV between normal-weight and overweight people. The value of TV was significantly higher, and the TSH serum concentration was significantly lower among obese and overweight people taken together than among people with normal weight or underweight taken together. We did not find any significant differences in the biochemical parameters of thyroid function depending on the presence of MetS. It should be emphasized, however, that in the studied population, the TV differed significantly between women and men, which may affect the results.

Xiao et al. conducted a study in China on a large group of teenagers. It was found that among boys, the TV in obese and overweight people was significantly higher than that in people with a normal body weight, while in girls, the TV in overweight people was significantly higher than that in people with a normal body weight. In our study, we did not analyze the sexes separately due to too few participants, but we also found that the TV in overweight people was significantly higher than that in people with a normal body weight. Moreover, we showed that when considering overweight and obese people together, the TV among these people is significantly higher than that in underweight and normal-weight people. Moreover, Xiao et al. indicated that the TV increases significantly with the increase in the number of components of MetS [20]. Due to the small size of the study group, we did not analyze the relationships examined according to the number of components of MetS, but in our population, the TV was significantly higher among people with MetS compared to people without MetS. Another study conducted in China (Guo et al.) but in the adult population also showed a positive correlation between the TV and the presence of MetS [21]. Similarly, according to the results obtained by a team of researchers from Turkey, the TV is significantly higher in patients with MetS compared to people without MetS [22]. According to the results obtained by Su et al., waist circumference significantly correlates with TV, which is also confirmed by the results obtained in our study [23].

According to the results obtained by Lass et al., the TV and TSH concentrations are significantly higher among overweight people compared to people with a normal body weight. We obtained similar results when comparing the TV of overweight and obese people to that of normal and underweight people, although in our study population, the TSH serum concentration was significantly lower in overweight and obese patients when compared to underweight or normal-weight people. Unlike the research conducted by the authors of the cited paper, we studied adults, not children, and we did not find any significant differences in the FT3 concentration [24]. Although, according to the results of many studies conducted so far, TSH serum concentration is positively correlated with BMI [25,26], the data on this subject are not fully clear. Manji et al. found no difference in the serum TSH or FT4 between lean and obese euthyroid subjects [27]. Interestingly, Velluzzi et al. did not find any relationship between the TSH serum concentration and BMI, except for women with anti-thyroid autoantibodies [28]. It is noteworthy that although in our population, the TSH concentration is significantly lower in the group of overweight or obese people compared to underweight or normal-weight people, there is no significant correlation between the TSH concentration and the BMI value in the entire study population.

Aldhafiri et al. presented the results of a study performed in Saudi Arabia involving 100 men with MetS and 100 men without MetS. Similarly to our results, there were no significant differences in the serum FT3 and FT4 levels depending on the diagnosis of MetS. However, unlike in our population, the serum TSH concentration was found to be significantly higher among people with MetS. It should be noted, however, that the cited publication also included people with overt thyroid disease, whereas our study included only euthyroid people and did not include people with diagnosed thyroid disease. Moreover, the authors of the mentioned publication did not take into account the TV in the presented results [29].

Kommareddy et al. conducted a cross-sectional study on a large group of overweight or obese euthyroid people. Similarly to our results, the authors of the mentioned study did not find a significant difference between the TSH concentration in the serum between people with MetS and people without MetS [30]. On the other hand, according to Waring et al., in fully adjusted models, each unit increase in TSH was associated with a significant increase in the prevalence of MetS, even when only euthyroid patients were taken into consideration (OR 1.16; 95% CI: 1.03–1.30; *p* = 0.02). Moreover, it was shown that although subclinical hypothyroidism with a serum TSH level of >10 µIU/mL is associated with an increased incidence of MetS (OR 2.3; 95% CI: 1.0–5.0), it is not associated with a significantly increased incidence of MetS in a 6-year follow-up period (OR 2.2; 95% CI: 0.6–7.5) [31].

The relationship between increased body weight and the hypothalamic–pituitary–thyroid axis is bidirectional. Thyroid hormones are involved in the control of metabolism, and the mechanisms by which increased body weight affects thyroid function are discussed in the literature. Among these pathophysiological mechanisms, attention is drawn to the role of adipose tissue dysfunction, hyperleptinemia, changes in deiodinase activity, central and peripheral resistance to thyroid hormones, insulin resistance, and chronic low-grade inflammation [12,32,33,34,35].

Adipose tissue dysfunction is characterized by an imbalance between proinflammatory and anti-inflammatory adipokine activity, which is associated with chronic low-grade inflammation, hypoxia, and increased oxidative stress. Adipose tissue dysfunction is the crucial mechanism leading to the development of obesity-related metabolic disturbances, such as insulin resistance, hypertension, and dyslipidemia [36,37]. Leptin is considered one of the main factors responsible for the impact of excessive body weight on the function of the hypothalamic–pituitary–thyroid axis [38]. It was found that leptin increases the synthesis of thyrotropin-releasing hormone (TRH). The mechanisms explaining this phenomenon include, among others, activation of the STAT3 transcription factor [39] and activation of the synthesis of α-melanocyte stimulating hormone (α-MSH) [40]. However, according to the results presented by Adamczewska et al., among obese children with idiopathic short stature, there was no significant relationship between the leptin serum concentration and the TSH serum concentration, which shows that pathophysiological phenomena related to the functioning of the hypothalamic–pituitary–thyroid axis in patients with excessive bodyweight are not yet fully understood, and further research is necessary [41].

### Strengths and Limitations of the Study

The presented study has some limitations. First of all, due to the nature of the study (cross-sectional), conclusions can only be drawn about the existence of certain relationships at a specific point in time. However, no conclusions can be drawn about possible cause-and-effect relationships. Another disadvantage is the relatively small study group, so it would be worth repeating the study for a larger population. Due to the small numbers, underweight people were not included in the variance analysis according to the BMI categories. Moreover, in the case of a larger number of people with MetS, additional analyses could be performed according to the constellation of MetS components present in specific people, because the population of people with MetS is not homogeneous. Another limitation is the lack of antibody tests used in the diagnosis of thyroid diseases, because it cannot be ruled out that the presence of antibodies, even without clinically evident thyroid disease, may influence the described relationships.

This study also has some strengths. The most significant strength of our study is the simultaneous consideration of TV, the biochemical assessments of thyroid function (TSH, FT3, and FT4 simultaneously), anthropometric measurements, and BCAs. All data were collected from the patients during their hospitalization, which means that the information about the patients came from current assessments and was not based on interview data. There were also very few missing data in the collected material, which is important considering the retrospective nature of the study.

## 5. Conclusions

Our results show that among euthyroid people, patients with MetS are characterized by a larger TV compared to that of people without MetS, and overweight and obese people are characterized by significantly higher values of TV and a significantly lower TSH serum concentration compared to underweight and normal-weight people. Moreover, among euthyroid people, there are significant correlations between the TV and the serum FT3 concentration, as well as some parameters of BCAs.

In the available literature, significantly high variability can be observed in the conclusions drawn from similar studies conducted so far, which may be related to the differences in the inclusion criteria for individual studies. Moreover, the analyzed differences in the values of thyroid morphology and function parameters, although statistically significant, are often so small that they may be disturbed by some small random variability in the examined parameters.

Further prospective studies are necessary to determine the cause-and-effect relationships between thyroid morphology and thyroid function parameters, metabolic health, and BCAs. Moreover, further research is necessary to better understand the pathophysiological mechanisms leading to the interrelationships between body weight and composition, the presence of features of metabolic syndrome, and the morphology and function of the thyroid gland.

## Figures and Tables

**Table 1:** Diagnostic criteria for metabolic syndrome (MetS) [19].

(1) Central obesity, defined as increased waist circumference (≥94 cm in men; ≥80 cm in women).
(2) Systolic blood pressure of ≥130 mmHg or diastolic blood pressure of ≥85 mmHg, or antihypertensive drug use by a patient with diagnosed arterial hypertension.
(3) Triglyceride serum concentration of ≥150 mg/dL (1.7 mmol/L) or pharmacological treatment of this lipid disorder.
(4) High-density lipoprotein cholesterol of <40 mg/dL in men or <50 mg/dL in women or pharmacological treatment for this lipid disorder.
(5) Fasting venous blood plasma glucose concentration of ≥100 mg/dL or pharmacological treatment of diagnosed carbohydrate metabolism disorders.

**Table 2:** Descriptive statistics of parameters for assessing thyroid morphology and function.

Parameter	N	Mean (SD)/ Median (Q1; Q3)	Range
TSH (µIU/mL)	45	1.46 (0.7)	0.298–3.06
FT4 (ng/dL)	45	1.26 (0.18)	0.9–1.7
FT3 (pg/mL)	45	3.08 (0.55)	1.8–4.5
TV (mL)	44	12.6 (9.95; 16.3)	6.6–26.6

Abbreviations: TSH—thyrotropin; FT4—free thyroxine; FT3—free triiodothyronine; TV—thyroid volume; SD—standard deviation; Q1—the first quartile; Q3—the third quartile.

**Table 3:** Descriptive statistics of anthropometric parameters and body composition analysis (BCA) results.

Parameter	N	Mean (SD)/Median (Q1; Q3)	Range
BMI (kg/m^2^)	45	25.84 (4.88)	16.40–37.47
Waist circumference (cm)	45	89.24 (14.26)	61.0–117.0
WHR	45	0.88 (0.08)	0.71–1.05
WtHR	45	0.53 (0.09)	0.37–0.69
Fat mass (kg)	45	20.66 (8.18)	4.4–36.0
Fat percentage (%)	45	27.10 (7.75)	7.3–41.0
Fat-free mass (kg)	45	53.75 (9.63)	38.2–73.0
Bone mass (kg)	45	2.71 (0.45)	2.0–3.6
Muscle mass (kg)	45	51.04 (9.18)	36.2–69.4
Muscle mass (%)	45	69.22 (7.37)	56.0–88.2
Skeletal muscle mass (kg)	45	25.5 (23.0–33.8)	18.6; 42.2
Total body water (kg)	45	37.90 (7.65)	25.6–57.2
Total body water (%)	45	50.24 (6.85)	31.1–65.2
Extracellular-to-total body water ratio (%)	45	43.0 (41.3; 45.7)	39.7–51.2
Visceral fat rating	45	7.24 (3.91)	1–16
Metabolic age	45	43.49 (14.75)	12–71
Basic metabolic rate (kcal)	45	1591.22 (276.63)	1127.0–2232.0

Abbreviations: BMI—body mass index; WHR—waist–hip ratio; WtHR—waist–height ratio; SD—standard deviation; Q1—the first quartile; Q3—the third quartile.

**Table 4:** Distribution of study participants by body mass index (BMI) category.

BMI Category	N (%)
Underweight	4 (8.9%)
Normal weight	15 (33.3%)
Overweight	15 (33.3%)
Obesity	11 (24.4%)

**Table 5:** The results of the Spearman’s rank correlation test between the parameters of thyroid function assessment, anthropometric parameters, and BCA.

Parameter	TSH		FT3		FT4		Thyroid Volume	
	*R*	*p*	*R*	*p*	*R*	*p*	*R*	*p*
BMI	−0.187	0.218	0.35	0.018	−0.069	0.654	0.247	0.107
Waist circumference	−0.182	0.231	0.381	0.01	−0.091	0.551	0.401	0.007
WHR	−0.044	0.776	0.305	0.042	−0.067	0.66	0.264	0.084
WtHR	−0.186	0.222	0.391	0.008	−0.165	0.279	0.25	0.102
Fat mass	−0.082	0.591	0.252	0.095	−0.164	0.281	0.104	0.502
Fat percentage	−0.015	0.923	0.151	0.322	−0.242	0.11	−0.181	0.24
Fat-free mass	−0.177	0.246	0.337	0.024	0.26	0.084	0.621	<0.001
Bone mass	−0.175	0.251	0.327	0.028	0.25	0.09	0.631	<0.001
Muscle mass	−0.177	0.246	0.337	0.024	0.26	0.084	0.621	<0.001
Muscle percentage	0.008	0.96	−0.143	0.348	0.245	0.105	0.193	0.21
Skeletal muscle mass	−0.07	0.647	0.31	0.04	0.111	0.466	0.552	<0.001
Total body water mass	−0.217	0.152	0.297	0.047	0.256	0.09	0.56	<0.001
Total body water percentage	0.117	0.44	−0.14	0.356	0.034	0.822	0.179	0.246
Extracellular-to-total body water ratio	−0.094	0.54	−0.079	0.61	−0.055	0.721	−0.274	0.072
Visceral fat rating	−0.228	0.133	0.152	0.317	−0.125	0.413	0.386	0.01
Metabolic age	−0.21	0.171	0.06	0.693	−0.169	0.266	0.183	0.234
Basic metabolic rate	−0.174	0.254	0.346	0.02	0.231	0.126	0.593	<0.001

Abbreviations: BMI—body mass index; WHR—waist–hip ratio; WtHR—waist-to-height ratio; TSH—thyrotropin; FT3—free triiodothyronine; FT4—free thyroxine; R—Spearman’s rank correlation coefficient. Statistically significant correlations are marked in red.

**Table 6:** Differences in body composition analysis (BCA) parameters and thyroid volume (TV) and function between people with and without metabolic syndrome (MetS).

Parameter	Patients with MetS		Patients without MetS		*p*
	N	Mean (SD)/Median (Q1; Q3)	N	Mean (SD)/Median (Q1; Q3)	
Age (years)	13	53.52 (49.74; 57.99)	32	48.72 (31.63; 54.83)	0.030 **
BMI (kg/m^2^)	13	28.25 (4.12)	32	24.86 (4.89)	0.033 *
Waist circumference	13	98.92 (11.51)	32	85.31 (13.49)	0.003 *
WHR	13	0.94 (0.07)	32	0.86 (0.08)	0.004 *
WtHR	13	0.57 (0.06)	32	0.51 (0.09)	0.024 *
Fat mass (kg)	13	23.75 (7.39)	32	19.41 (8.26)	0.11 *
Fat percentage (%)	13	27.65 (5.96)	32	26.88 (8.45)	0.77 *
Fat-free mass (kg)	13	60.73 (8.74)	32	50.91 (8.56)	0.001 *
Bone mass (kg)	13	3.02 (0.39)	32	2.58 (0.41)	0.002 *
Muscle mass (kg)	13	57.71 (8.35)	32	48.33 (8.16)	0.001 *
Muscle mass (%)	13	68.73 (5.66)	32	69.41 (8.04)	0.78 *
Skeletal muscle mass (kg)	13	32.8 (26.7; 36.2)	32	24.2 (22.9; 31.4)	0.02 **
Total body water (kg)	13	42.3 (6.98)	32	36.1 (7.27)	0.012 *
Total body water (%)	13	50.37 (5.73)	32	50.18 (7.34)	0.94 *
Extracellular-to-total body water ratio	13	42.3 (41.1; 45.7)	32	43.1 (41.3; 45.7)	0.83 **
Visceral fat rating	13	10.39 (2.81)	32	5.97 (3.58)	<0.001 *
Metabolic age	13	54.31 (8.0)	32	39.1 (14.67)	0.001 *
Basic metabolic rate (kcal)	13	1783.62 (253.82)	32	1513.06 (248.69)	0.002 *
TSH (µIU/mL)	13	1.43 (0.41)	32	1.47 (0.8)	0.85 *
FT4 (ng/dL)	13	1.21 (0.14)	32	1.28 (0.19)	0.26 *
FT3 (pg/mL)	13	3.16 (0.41)	32	3.05 (0.6)	0.55 *
TV (mL)	13	15.4 (13.0; 21.5)	31	11.7 (8.6; 15.5)	0.025 **

Abbreviations: MetS—metabolic syndrome; SD—standard deviation; N—number of subjects; Q1—first quartile; Q3—third quartile; * *p*-value according to Student’s *t*-test; ** *p*-value according to the Mann–Whitney U test; BMI—body mass index; WHR—waist-to-hip ratio; WtHR—waist-to-height ratio; TSH—thyrotropin; FT4—free thyroxine; FT3—free triiodothyronine; TV—thyroid volume.

**Table 7:** Comparison of individual BMI categories in terms of thyroid volume and biochemical parameters assessing thyroid function using the ANOVA test or the Kruskal–Wallis test.

Parameter	Normal Weight		Overweight		Obesity		*p*
	N	Mean (SD)/Median (Q1; Q3)	N	Mean (SD)/Median (Q1; Q3)	N	Mean (SD)/Median (Q1; Q3)	
TSH (µIU/mL)	15	2.02 (1.05; 2.57)	15	1.11 (0.70; 1.41)	11	1.36 (1.04; 1.63)	0.063 *
FT3 (pg/mL)	15	3.14 (0.47)	15	2.95 (0.44)	11	3.44 (0.63)	0.064 **
fT4 (ng/dL)	15	1.29 (0.19)	15	1.21 (0.17)	11	1.27 (0.16)	0.444 **
TV (mL)	15	10.6 (8.3; 13.1)	15	13.0 (11.4; 21.8)	10	14.3 (9.3; 16.5)	0.026 *

Abbreviations: TSH—thyrotropin; FT3—free triiodothyronine; FT4—free thyroxine; SD—standard deviation; N—number of subjects; Q1—first quartile; Q3—third quartile; * *p*-value according to the Kruskal–Wallis test; ** *p*-value according to the ANOVA test.

**Table 8:** Comparison of normal-weight/underweight and overweight/obesity patients in terms of thyroid volume (TV) and biochemical parameters assessing thyroid function.

Parameter	BMI < 25.0 kg/m^2^		BMI ≥ 25.0 kg/m^2^		*p*
	N	Mean (SD)/Median (Q1; Q3)	N	Mean (SD)/Median (Q1; Q3)	
TSH (µIU/mL)	19	1.54 (1.1; 2.48)	26	1.21 (0.84; 1.44)	0.023 *
FT3 (pg/mL)	19	2.98 (0.52)	26	3.15 (0.57)	0.32 **
FT4 (ng/dL)	19	1.28 (0.20)	26	1.24 (0.17)	0.46 **
TV (mL)	19	10.9 (8.3; 13.1)	25	13.2 (11.4; 21.2)	0.015 *

Abbreviations: TSH—thyrotropin; FT3—free triiodothyronine; FT4—free thyroxine; SD—standard deviation; N—number of subjects; Q1—first quartile; Q3—third quartile; (*)—*p*-value according to the Mann–Whitney U test; (**)—*p*-value according to Student’s *t*-test.

## Data Availability

The data presented in this study are available upon request from the corresponding author.
